# The possibility of mutations of RAS signaling genes and/or *TP53* in combination as a negative prognostic impact on pathological stage I non‐small cell lung cancer

**DOI:** 10.1002/cam4.6535

**Published:** 2023-09-15

**Authors:** Takayuki Honda, Katsutoshi Seto, Satoshi Endo, Akira Takemoto, Kousuke Tanimoto, Masashi Kobayashi, Masatake Kitano, Rie Sakakibara, Takahiro Mitsumura, Hironori Ishibashi, Johji Inazawa, Toshihiro Tanaka, Yasunari Miyazaki, Kenichi Okubo

**Affiliations:** ^1^ Department of Respiratory Medicine Tokyo Medical and Dental University Bunkyo‐ku Japan; ^2^ Department of Thoracic Surgery Tokyo Medical and Dental University Bunkyo‐ku Japan; ^3^ Soka Municipal Hospital Soka‐shi Japan; ^4^ Bioresource Research Center Tokyo Medical and Dental University Bunkyo‐ku Japan; ^5^ Research Core Tokyo Medical and Dental University Bunkyo‐ku Japan; ^6^ Department of Thoracic Surgery Kurashiki Central Hospital Kurashiki Japan

**Keywords:** non‐small cell lung cancer, pathological stage I, RAS signaling genes, *TP53*

## Abstract

**Background:**

The recurrence rate of non‐small cell lung cancer (NSCLC) is as high as 30%, even in the cancer with pathological stage I disease. Therefore, identifying factors predictive of high‐risk pathological recurrence is important. However, few studies have examined the genetic status of these tumors and its relationship to prognosis.

**Materials and Methods:**

A cohort of 328 cases of primary lung cancer that underwent complete resection at Tokyo Medical and Dental University (TMDU) was screened for 440 cancer‐associated genes using panel testing. Further analyses included 92 cases of pathological stage I NSCLC who did not receive adjuvant chemotherapy. Ridge regression was performed to identify association studies mutational status and postoperative recurrence. These data were then validated using clinical and genetic data from 56 patients in The Cancer Genome Atlas (TCGA).

**Results:**

Mutations in *TP53*, RAS signaling genes *KRAS* and *HRAS*, and *EGFR* were recurrently detected. Ridge regression analysis relevant to recurrence, as well as survival analysis, performed using data from the TMDU cohort revealed significantly shorter relapse‐free survival (RFS) for patients with RAS signaling or *TP53* gene mutations than for those without (log‐rank test, *p* = 0.00090). This statistical trend was also suggested in the TCGA cohort (log‐rank test, *p* = 0.10).

**Conclusion:**

Mutations in RAS signaling genes and/or *TP53* could be useful for the prediction of shorter RFS of patients with stage I NSCLC.

## 1 INTRODUCTION

Lung cancer is a leading cause of cancer‐related death worldwide.^1^ One possible reason for the poor prognosis is high rates of recurrence after surgery (30%–40%), even in those with pathological stage I non‐small cell lung cancer (NSCLC).^2^, ^3^ Therefore, the use of adjuvant therapy (comprising mainly of platinum) for postoperative NSCLC patients at high‐risk of recurrence should be considered to improve survival.^4^ Recent clinical studies show that adjuvant therapies based on molecular‐targeted agents and immune checkpoint inhibitors also have a positive impact on survival of postsurgical NSCLC patients.^5^, ^6^

However, the indications for adjuvant therapy for stage I NSCLC are still not straightforward because the effects of adjuvant chemotherapy at this stage are controversial.^4^, ^7^ One possible reason is the heterogeneity of stage I NSCLC. For example, lung adenocarcinomas (LADCs) with the lepidic‐predominant histological subtype in which *EGFR* is frequently mutated^8^ show better prognosis after surgery (up to 100% relapse‐free survival) than LADCs with the papillary and solid subtypes.^9^ Therefore, identifying stage I NSCLC patients at high‐risk of relapse is important because we could maximize survival by selecting optimal candidates for adjuvant therapy.^10^

Cancer panel testing using next‐generation sequencing is used frequently to identify druggable gene mutations^11^, ^12^ and to enable oncologists to access representative genomic information for a tumor, even if it lacks druggable gene aberrations. Driver oncogene mutations are closely related to the clinicopathological phenotype of NSCLC^8^; as such, they might have prognostic impact on the disease course.^13^, ^14^ These facts suggest that not only clinicopathological analysis, but also cancer‐related gene mutations in a tumor, could help us to predict disease course, e.g., the likelihood of relapse after surgery. Because few reports have explored the association between gene mutations and relapse‐free survival (RFS) after surgery, we aimed to examine the prognostic impact (i.e., RFS) of screening for cancer‐related gene mutations using cancer panel testing, especially in patients with pathological stage I NSCLC.

## 2 MATERIALS AND METHODS

### 2.1 Patient selection

A total of 328 consecutive lung cancer patients aged ≥20 years, who underwent surgical resection between 2014 and 2019 at Tokyo Medical and Dental University (TMDU), were screened for 440 cancer‐related genes. In addition, data from 92 patients with pathological stage I NSCLC who did not receive adjuvant chemotherapy were extracted from the 328 surgically treated lung cancer patients (the TMDU cohort). All patients were staged according to the seventh edition of the TMN classification^15^; this is because most studies of the effects of adjuvant therapy are based on this edition.^4^ The inclusion criteria were as follows: curative surgery with lobectomy, but without neoadjuvant chemotherapy or radiotherapy, and a definitive histologic diagnosis of NSCLC. Clinical data collected from the medical records of each patient included age, gender, Eastern Cooperative Group Performance Status (ECOG‐PS), adjuvant therapy, pathological findings, and RFS. The study was conducted in accordance with the tenets of the Declaration of Helsinki (revised in 2013) and was approved by the institutional review board of TMDU (G2019‐005, M2022‐018).

### 2.2 Pathologic examination

Pathological reviews based on the WHO classification^16^ were performed by an expert pathologist (AT). Lymphatic and vascular invasion were assessed by immunohistochemistry to detect D2‐40 (lymphatic ducts) and elastic Van Gieson staining to detect elastic fibers in the vessels, respectively. Lymphatic invasion and vascular invasion were deemed positive if a malignant neoplasm had spread through the lymphatic system or penetrated the vasculature, respectively. Pleural invasion was defined as positive invasion beyond the elastic layer and into the visceral pleural surface.

### 2.3 Detection and analysis of cancer‐related genomic mutations

All surgically resected samples were fixed with 10% neutral buffered formalin for 2 days immediately after surgery and paraffin‐embedded. Formalin‐fixed paraffin‐embedded (FFPE) tissues were stored at room temperature in the Bioresource Research Center of TMDU, and genomic DNA of tumor was extracted from the FFPE within 6 years in this study.

This study used genomic information analyzed using ACTOnco®+ (ActMed Co., Ltd.) and preserved in the Bioresource Research Center of TMDU. The detailed sequencing and data analysis protocols are described in the ACTOnco®+ report supplied by ACT Genomics Co., Ltd. Briefly, genomic DNA from cancer cells underwent in‐house quality check and was amplified using four pools of primer pairs targeting the coding exons of 440 cancer‐related genes (Table S1). The amplicons were ligated to barcoded adaptors. The quality and quantity of the amplified library were determined using a fragment analyzer (AATI) and Qubit (Invitrogen). Subsequently, barcoded libraries were conjugated to sequencing beads by emulsion PCR and then enriched using an Ion Chef system (Thermo Fisher Scientific) according to the Ion PI Hi‐Q Chef Kit protocol (Thermo Fisher Scientific) or the Ion 540 Kit‐Chef protocol (Thermo Fisher Scientific). Sequencing was performed on an Ion Proton or Ion S5 sequencer (Thermo Fisher Scientific).

Raw reads generated by the sequencer were mapped onto the hg19 reference genome using Ion Torrent Suite (version 5.10). Coverage depth was calculated using the Torrent Coverage Analysis plug‐in. Single nucleotide variants and short insertions/deletions (INDELs) were identified using the Torrent Variant Caller plug‐in (version 5.10). The coverage was down‐sampled to 4000. Variant Effect Predictor (VEP; version 88) was used to annotate every variant using databases from COSMIC v.92; dbSNP 151. Variants recorded in ToMMo 8.3 K or genomAD v2.1 were disregarded as polymorphisms and excluded from further analysis. Variants with coverage ≥25 and allele frequency ≥5% and actionable variants with allele frequency ≥2% were retained. Variants reported in the Genome Aggregation database r2.0.2 and with minor allele frequency (MAF) >1% were considered to be polymorphisms. An in‐house database of ACT Genomics was used to determine technical errors. For subsequent analyses, the variants included in dbSNP or COSMIC were used as clinically actionable and biologically significant variants. The results of gene mutations were displayed using “maftools” software (R, version 4.1‐2).

### 2.4 External datasets from a Caucasian population extracted from The Cancer Genome Atlas

To validate the results obtained from the TMDU cohort, clinical and genomic data from NSCLC patients in the “TRACERx” study^17^, ^18^ of The Cancer Genome Atlas (TCGA) were obtained from cBioportal (https://www.cbioportal.org/study/summary?id=nsclc_tracerx_2017. Accessed May 16, 2022).

### 2.5 Statistical analysis

Ridge regression, which is a machine learning model used when the regression data to be analyzed are significantly affected by multicollinearity, was applied to examine the association between gene mutations and RFS. Ridge regression uses L2 regularization to penalize residuals when the parameters of a regression model are being learned; it is augmented with 8‐fold cross validation for internal validation and can be used to estimate the significance of predictors based on penalizing insignificant predictors. R package “glmnet” software (R, version 4.1‐2) was used to perform this regression analysis.

Summary statistics related to clinicopathology and gene mutations were applied. The chi‐square test, or Fisher's exact test, was used to compare categorical variables. The Mann–Whitney *U* test was used to compare numerical variables. The Kaplan–Meier method was used for survival analysis. Statistical analysis was performed using R package (version 4.1‐2). *P‐*values <0.05 and <0.10 were used to denote significance and trend, respectively.

## 3 RESULTS

### 3.1 Clinical and genomic characteristics of the Tokyo Medical and Dental University cohort

Ninety‐two patients with pathological stage I NSCLC were recruited. Patient characteristics are shown in Table 1. The TMDU cohort included 60 men (65.2%) and 32 women (34.8%), with a median age of 73 years (range, 29–87). Most patients in the TMDU cohort had a smoking history (Ever, *n* = 56 [60.9%]; Current, *n* = 8 [8.7%]). Fifty‐seven patients (61.9%) had LADC histology, and 33 (35.9%) had squamous cell carcinoma (LSQC) histology. Lymphatic, vascular, and visceral pleural invasion, all of which are risk factors for relapse of stage I NSCLC,^10^ were positive in three (3.3%), 24 (26.1%), and 20 (21.7%) patients, respectively. In the TMDU cohort, nine patients (9.8%) had recurrence.

**TABLE 1:** Clinical characteristics of the Tokyo Medical and Dental University (TMDU) cohort.

Characteristics	TMDU cohort (*n* = 92)
Age	73 (range, 29–87)
Gender (%)	
Male	60 (65.2)
Female	32 (34.8)
Smoking History (%)	
Never	28 (30.4)
Ever	56 (60.9)
Current	8 (8.7)
ECOG‐PS	
0	91 (98.9)
1	1 (1.1)
Histology (%)	
Adenocarcinoma	57 (61.9)
Squamous cell carcinoma	33 (35.9)
NSCLC, NOS	2 (2.2)
T stage (%)	
T1a	51 (55.4)
T1b	8 (8.7)
T2a	33 (35.9)
Lymphatic invasion (%)	3 (3.3)
Vascular invasion (%)	24 (26.1)
Visceral pleural invasion (%)	20 (21.7)
Recurrence (%)	
No	83 (90.2)
Yes	9 (9.8)

Abbreviations: ECOG‐PS, Eastern Cooperative Oncology Group Performance Status; NOS, not otherwise specified; NSCLC, non‐small cell lung carcinoma.

**FIGURE 1:**
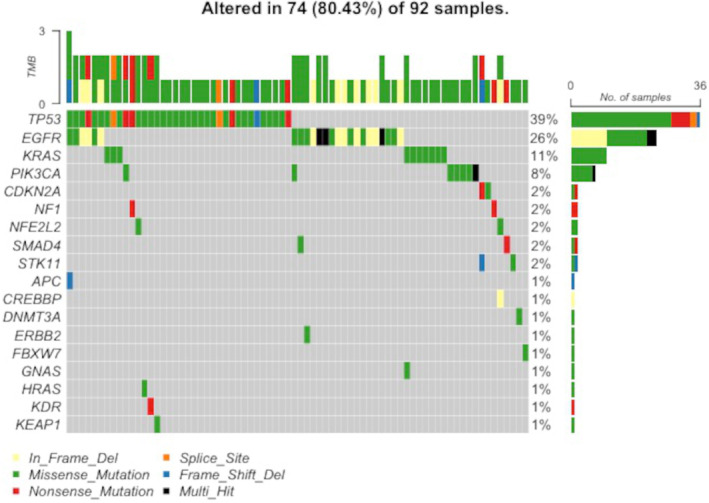
Gene mutations observed in the Tokyo Medical and Dental University with pathological stage I non‐small cell lung cancer are displayed. The oncoplot shows the genes according to descending order, and the colors of each bar indicate the type of mutation such as missense or insertion–deletion.

Cancer panel sequencing was performed, and Figure 1 shows the mutational landscape of the TMDU cohort. Eighteen genes were identified; the most common mutated gene showing partial duplication was *TP53* (*n* = 36, 39.1%), followed by *EGFR* (*n* = 24, 26.1%), *KRAS* (*n* = 10, 10.9%), and *PIK3CA* (*n* = 7, 7.6%). These data are in agreement with those reported in previous studies of LADC and LSQC in East Asian cohorts.^19^, ^20^

### 3.2 Identification of genomic mutations associated with recurrence after surgery

**FIGURE 2:**
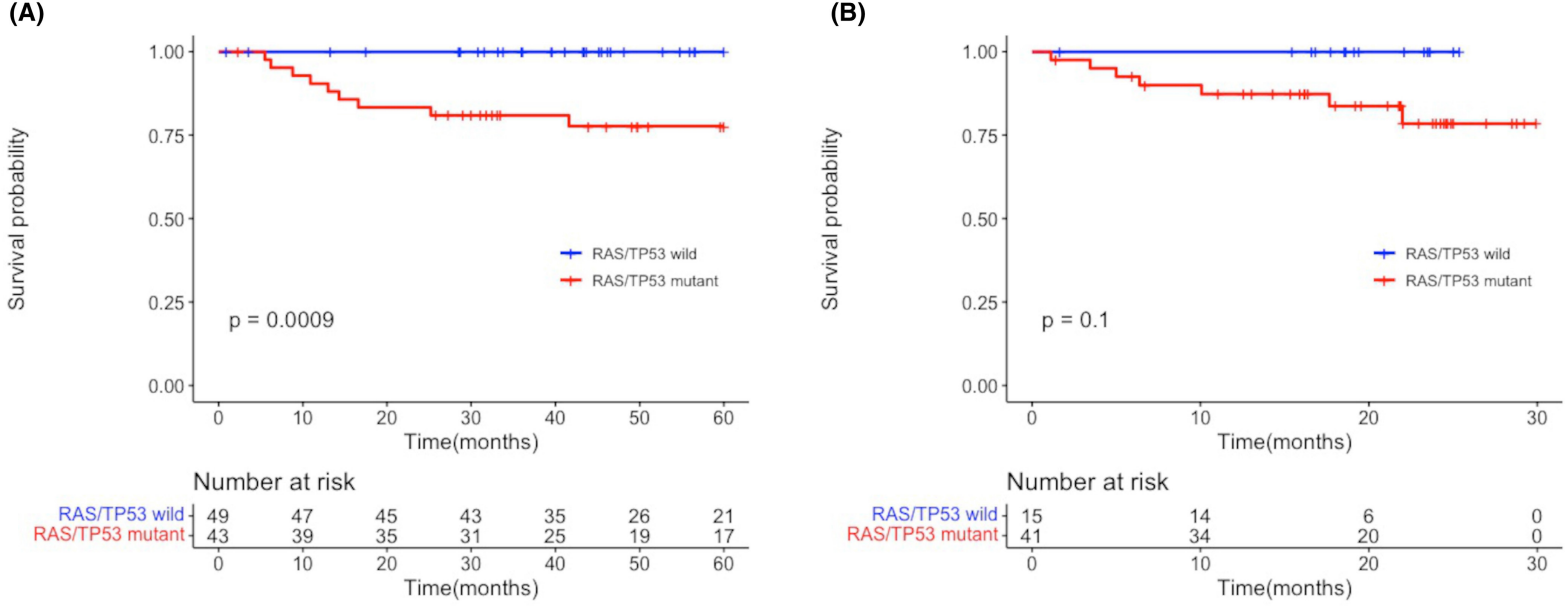
Recurrence‐free survival in the Tokyo Medical and Dental University cohort (A) and in the The Cancer Genome Atlas cohort (B) displayed using Kaplan–Meier curve. *p* Values derived from log‐rank test and the number at risk tables of each cohort are also displayed.

Association analysis was performed to identify candidate gene mutations associated with recurrence. Recurrently mutated genes (observed in two or more patients in the TMDU cohort) were selected and included in Ridge regression analysis. Of these, *KRAS* and/or *TP53* correlated positively with relapse events (Table S2). Survival analysis using the Kaplan–Meier method confirmed inferior survival of patients carrying *KRAS* and/or *TP53* mutations and superior survival of patients carrying the *EGFR* mutation (Figure S1A–C). Because evidence shows that accumulated gene mutations in the RAS signaling pathway lead to increased malignancy,^21^, ^22^ gene mutations in RAS signaling pathway (e.g., *KRAS* and *HRAS*) identified in the TMDU cohort could be associated with early recurrence after surgery. Thus, we divided patients into two groups according to RAS gene mutations and/or *TP53* mutations: a RAS/TP53‐mutant group and a RAS/TP53‐wild‐type (WT) group. Although there were no significant differences between the two groups with respect to potential prognostic factors for relapse (the exception was visceral pleural invasion)^10^ (Table 2, Figure S2A–B), the RAS/TP53‐mutant group showed significantly shorter RFS than the RAS/TP53‐WT group (log‐rank test; *p* = 0.00090; Figure 2A). Stratification according to visceral pleural invasion (to adjust for this confounding factor) confirmed the poor prognostic trend in the RAS/TP53‐mutant group (Figure S3).

**TABLE 2:** Potential prognostic factors between RAS/TP53 mutant and wild‐type (WT).

Characteristics	RAS/TP53‐mutant (*n* = 43)	RAS/TP53‐WT (*n* = 49)	*p* Value
Age	72	74	0.40
T stage (%)			
T1	23 (53.5)	36 (73.5)	0.08
T2a	20 (46.5)	13 (26.5)	
Histology			
Adenocarcinoma (%)	23 (53.5)	34 (69.4)	0.07
Squamous cell carcinoma (%)	20 (46.5)	13 (26.5)	
NOS	0	2 (4.1)	
Lymphatic invasion (%)	2 (4.7)	1 (2.0)	0.60
Vascular invasion (%)	12 (27.9)	13 (26.5)	0.89
Visceral pleural invasion (%)	14 (32.6)	6 (12.4)	0.02

*Note*: These variables are identified as prognostic factors for relapse‐free survival in ref.^10^ *p* Value were calculated using the chi‐squared test or Fisher's exact test or Mann–Whitney *U* test, where appropriate.

Abbreviation: NOS, not otherwise specified.

TCGA data from 56 stage I NSCLC patients without adjuvant chemotherapy were extracted and used as a Caucasian TCGA cohort. In this cohort, neither patients with RAS gene mutations nor with *TP53* did not show worse RFS compared to patients with each wild‐type alone (data not shown). However, the statistical trend in survival prognosis observed for the TMDU cohort was also suggested for the TCGA cohort (log‐rank test; *p* = 0.10; Figure 2B) when RAS signaling genes and/or *TP53* in combination.

## 4 DISCUSSION

This study aimed to investigate whether gene mutations have an impact on RFS of patients with stage I NSCLC not receiving adjuvant therapy. Some previous studies investigated the association between RFS of early‐stage NSCLC and hotspot mutation of *KRAS* and/or *TP53* obtained by resected tumor or noncancerous tissue located near resected tumor,^23^, ^24^ but the results were not necessarily in agreement. Our study using panel testing covering all exonic regions of these genes showed the practical utility of gene mutations of RAS signaling molecules and/or *TP53* in combination which were associated with a worse prognosis in the TMDU cohort and were suggested in the TCGA cohort.

Surgery is the best treatment for operable NSCLC. However, the prognosis of these patients is not satisfying due to the high frequency of postoperative recurrence. Indeed, 40% of patients in whom stage I NSCLC is resected completely experience recurrence, and the absolute risk of distant metastasis exceeds that of local recurrence at any stage.^3^ The predominant pattern of distant metastasis even for stage I NSCLC strongly suggests the presence of occult metastasis that escapes conventional staging.^25^ Therefore, systemic adjuvant therapy plays an important role in prolonging the survival of patients with surgically resected NSCLC. Although previous clinical studies show that addition of adjuvant chemotherapy, mainly using platinum, improves survival,^4^ the prognostic improvement achieved by adjuvant chemotherapy is not the same for stage “I” versus “II and III” NSCLCs; indeed, the effect of adjuvant chemotherapy seems to diminish the earlier the pathological stage becomes.^7^ A recent study showing the survival benefits of a PD‐L1 inhibitor as adjuvant therapy reported increased disease‐free survival of patients with PD‐L1‐positive stage II–IIIA NSCLC.^6^ In contrast to PD‐L1 inhibitors, adjuvant therapy using osimertinib, which is used postoperatively for *EGFR* mutation‐positive NSCLC patients, improved survival at any stage, including stage IB.^5^

One possible reason for the controversy surrounding adjuvant therapy for stage I NSCLC is the clinical heterogeneity of the cancer at this stage. A previous study showed an indivisible association between driver oncogene aberrations and pathological morphology of LADC, both of which may be related to prognosis.^8^ Because molecular‐targeted therapies seem to improve survival,^26^ the pathological classification of lung tumors has been refined through integrating the indivisible association.^27^ These findings suggest that genetic alterations in a tumor have the potential to predict disease course. In fact, genomic analysis of lung cancer using whole‐exome sequencing (WES) suggests that driver oncogenes might be associated with a poor prognosis.^14^ Pioneering scientists at the Memorial Sloan Kettering (MSK) Cancer Center, USA, developed an MSK‐IMPACT test capable of detecting alterations in more than 300 cancer‐associated genes with the hope of detecting drug responses; this panel test is now a standard for clinical sequencing.^28^ Easy accessibility to panel testing and evidence of successful adjuvant therapy using osimertinib might encourage relatively early genetic screening of surgically resected tumors before they recur.

In this study, we found that specific mutations of RAS signaling molecules and/or *TP53* in combination were associated negatively with RFS of patients with stage I NSCLC. RAS signaling pathway genes such as *KRAS* and *HRAS*, as well as *TP53*, are often mutated in solid tumors, including lung cancer with LADC and LSQC histology.^19^, ^20^, ^29^ Evidence suggests that gene dysregulation caused by these mutations promotes oncogenicity and endows tumors with malignant potential.^21^, ^22^ A previous clinicopathological study also showed LADC with *KRAS* mutation were more likely to invade visceral pleura than LADC without the mutation suggesting negative prognostic impact of *KRAS* mutation,^30^ though the frequency of visceral pleural invasion was not different between NSCLC with KRAS mutant (30%, 3/10) and KRAS‐WT (17/65; *p* = 0.45, Fisher's exact test) in the TMDU cohort. WES, followed by calculation of genomic status, reveals that alterations in non‐cancer‐associated genes such as *MUC4* are associated with RFS of stage I NSCLC.^31^ However, WES is not yet widely available in the clinic. Paradoxically, gene mutation data obtained by WES complicate identification of significantly mutated genes; in fact, the MSK‐IMPACT test, which is used for clinical sequencing, restrictedly consists of 100 cancer‐associated genes.^28^, ^32^ Our study was also performed using only gene mutation data available in a clinical setting. Adjuvant chemotherapy for high‐risk stage I NSCLC improves cancer‐specific survival.^10^ Our results may facilitate selection of suitable candidates for this type of therapy even though previous studies of adjuvant chemotherapy ranged from stage I to III NSCLC with *KRAS* and *TP53* co‐mutations could not prove clinical benefit,^33^, ^34^ while patients with these co‐mutations might have predictive value of immunotherapy^35^ through increased expression of PD‐L1 and mutation burden.^36^ NSCLC patients with these mutations might benefit from adjuvant therapy using only PD‐1 inhibitor.^37^

Our study has several limitations. First, the panel test did not consider the frequency of fusion gene aberrations such as *ALK*, *ROS1*, and *RET* in LADC. Second, our study population consisted of patients with stage I NSCLC (approximately 100 in the TMDU cohort and 60 in the TCGA cohort); however, the sample numbers are low, suggesting our results should be carefully interpreted because our data could not reduce to association of a single gene, thus making it difficult to characterize ethnic differences between Asian and Caucasian populations with respect to driver oncogene distribution, especially in LADC.^19^ Third, a verification study using TMDU patients could not be performed to confirm whether stage I NSCLC patients carrying RAS signaling and/or *TP 53* gene mutations in combination really do benefit from adjuvant therapy; this is because too few data for stage I NSCLC patients undergoing adjuvant chemotherapy were available.

In conclusion, the data presented herein suggest that stage I NSCLC patients with mutations of RAS signaling molecules and/or *TP53* in combination have shorter RFS than those without. This statistical trend was also suggested in a Caucasian cohort from the TCGA. Using genetic status to identify stage I NSCLC patients at high‐risk of recurrence would have the possibility of selecting those most likely to benefit from adjuvant therapy.

## AUTHOR CONTRIBUTIONS

**Takayuki Honda:** Conceptualization (lead); data curation (lead); formal analysis (lead); investigation (lead); methodology (lead); project administration (lead); resources (lead); software (lead); supervision (lead); validation (lead); visualization (lead); writing – original draft (lead); writing – review and editing (lead). **Katsutoshi Seto:** Conceptualization (equal); investigation (equal); visualization (equal); writing – original draft (equal). **Satoshi Endo:** Data curation (equal); investigation (equal); visualization (equal). **Akira Takemoto:** Data curation (equal); project administration (equal); resources (equal); software (equal); validation (equal). **Kousuke Tanimoto:** Data curation (equal); formal analysis (equal); investigation (equal); resources (equal); software (equal); supervision (equal). **Masashi Kobayashi:** Data curation (supporting); resources (supporting). **Masatake Kitano:** Data curation (supporting). **Rie Sakakibara:** Data curation (supporting). **Takahiro Mitsumura:** Data curation (equal). **Hironori Ishibashi:** Data curation (supporting); resources (supporting). **Johji Inazawa:** Funding acquisition (lead); project administration (lead); resources (equal); supervision (equal); writing – review and editing (equal). **Toshihiro Tanaka:** Funding acquisition (lead); project administration (lead); resources (equal); supervision (equal); writing – review and editing (equal). **Yasunari Miyazaki:** Project administration (equal); supervision (equal); writing – review and editing (equal). **Kenichi Okubo:** Data curation (equal); project administration (equal); resources (equal); supervision (equal); writing – review and editing (equal).

## ETHICS STATEMENT

All subjects participated voluntarily, and the participants provided their written informed consent to participate in this study. The study was conducted in accordance with the tenets of the Declaration of Helsinki (revised in 2013) and was approved by the institutional review board of TMDU (G2019‐005, M2022‐018).

## Supporting information


Figures S1–S3

Table S1–S2

Appendix S1

## Data Availability

Raw data were generated at Tokyo Medical and Dental University. Derived data supporting the findings of this study are available from the corresponding author Tokyo Medical and Dental University on request.
