# Supplementary material for: The possibility of mutations of RAS signaling genes and/or TP53 in combination as a negative prognostic impact on pathological stage I non‐small cell lung cancer
Source: Cancer Med. 2023 Sep 15;12(19):19406–13. doi: 10.1002/cam4.6535 (PMC10587933; doi:10.1002/cam4.6535)
Supplement: Supplementary file 1 — Figures S1–S3 [file CAM4-12-19406-s003.pptx]

## Slide 1
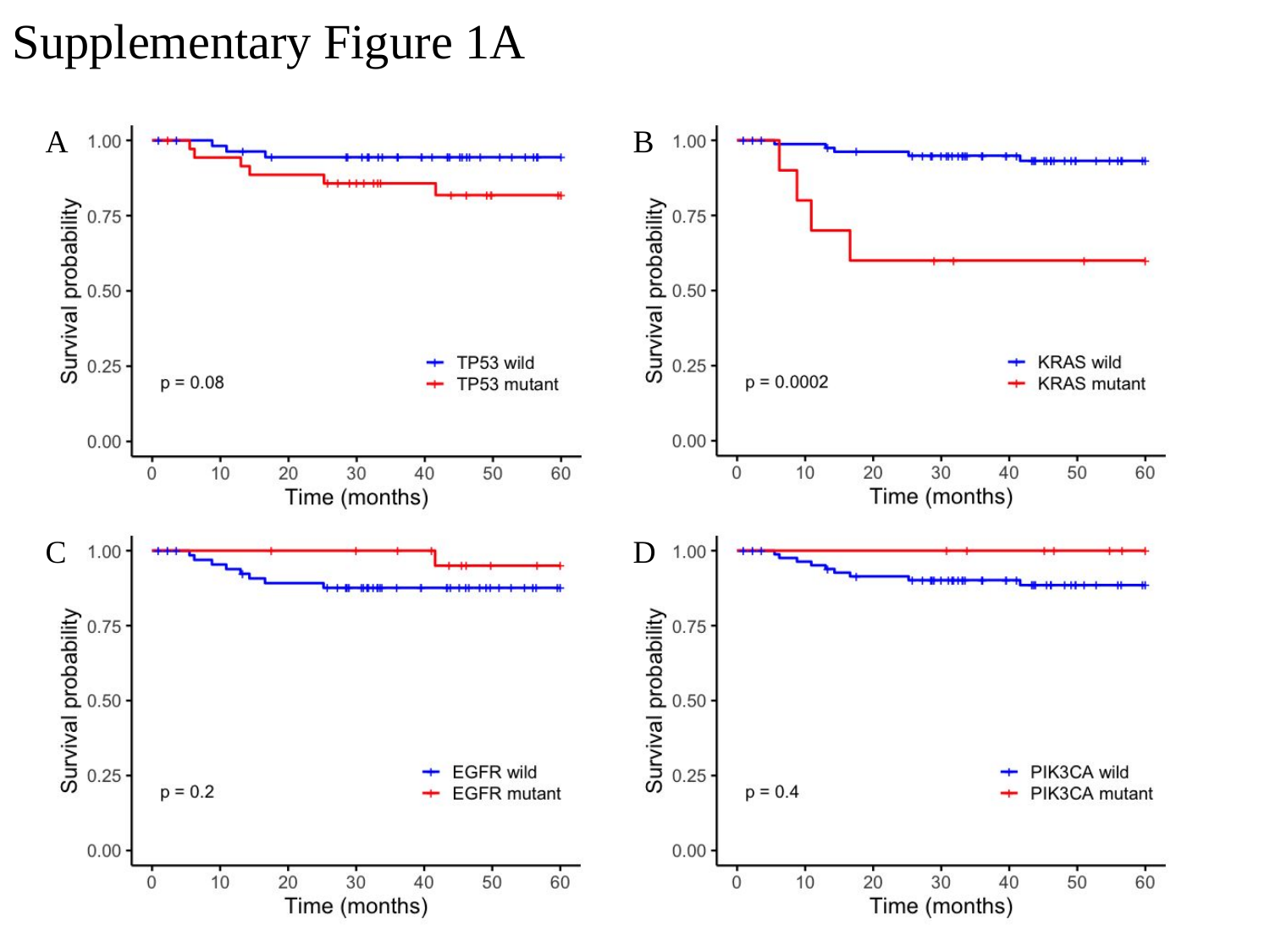

Supplementary Figure 1A
A
B
C
D

## Slide 2
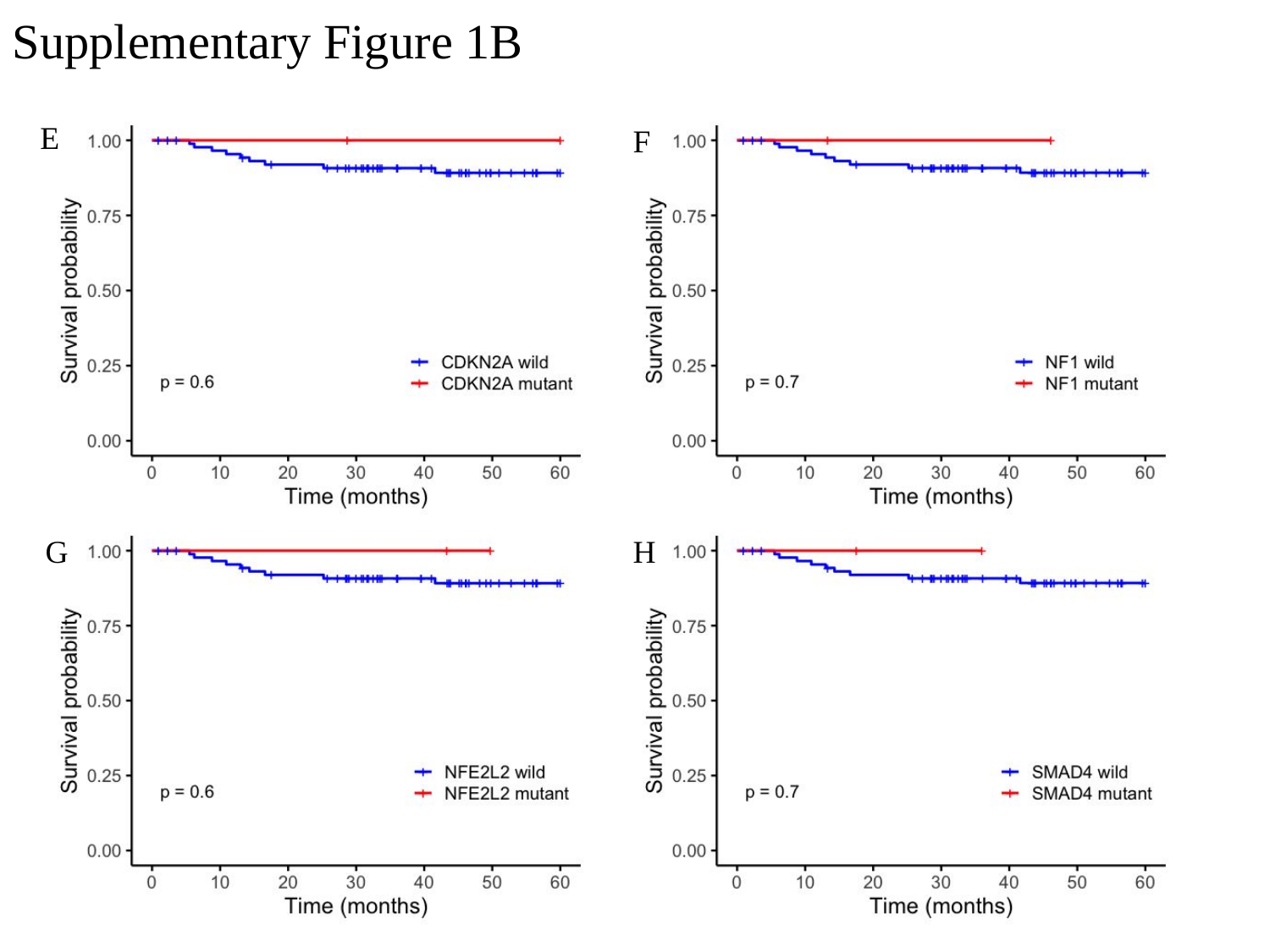

Supplementary Figure 1B
E
F
G
H

## Slide 3
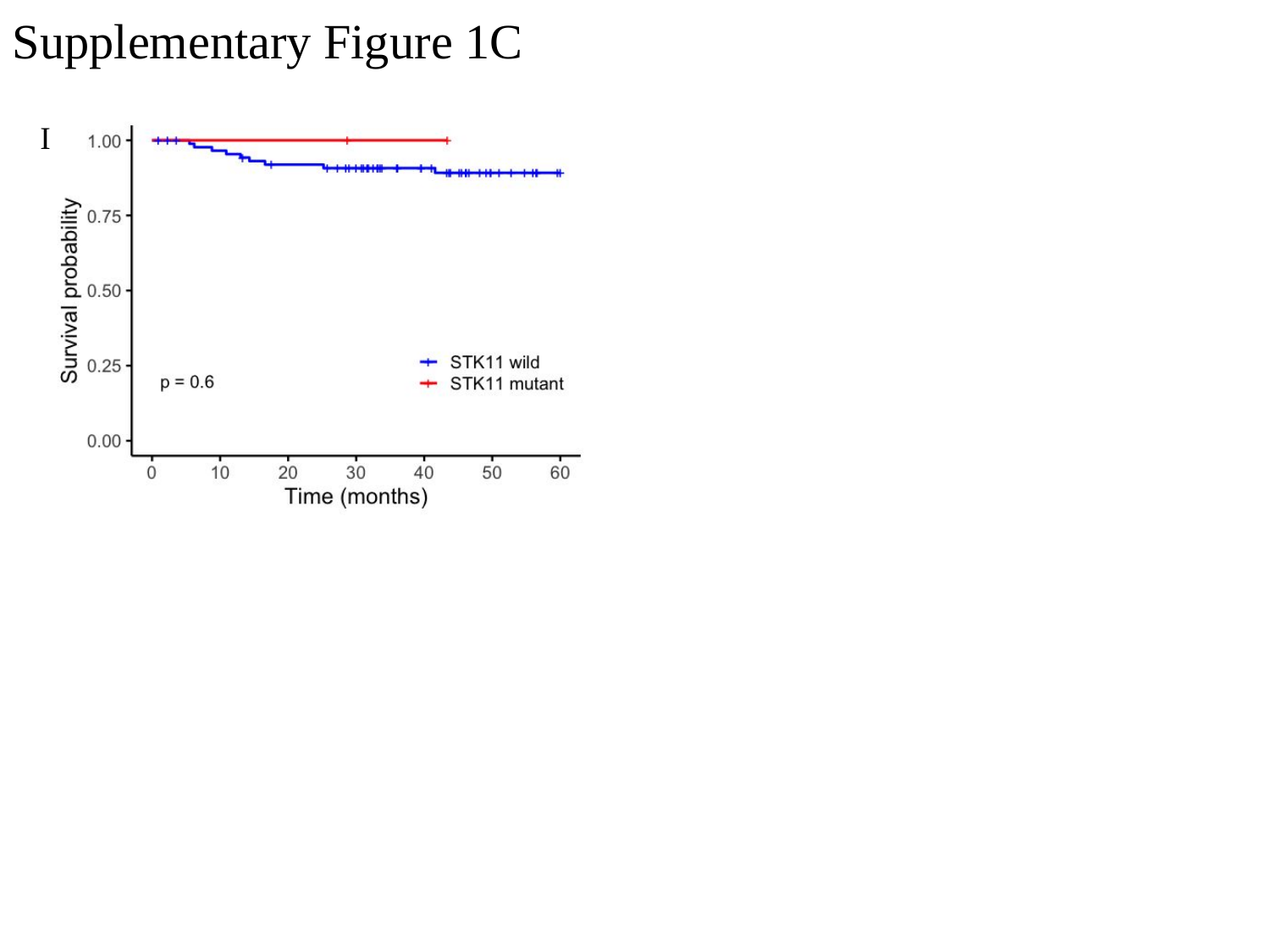

Supplementary Figure 1C
I

## Slide 4
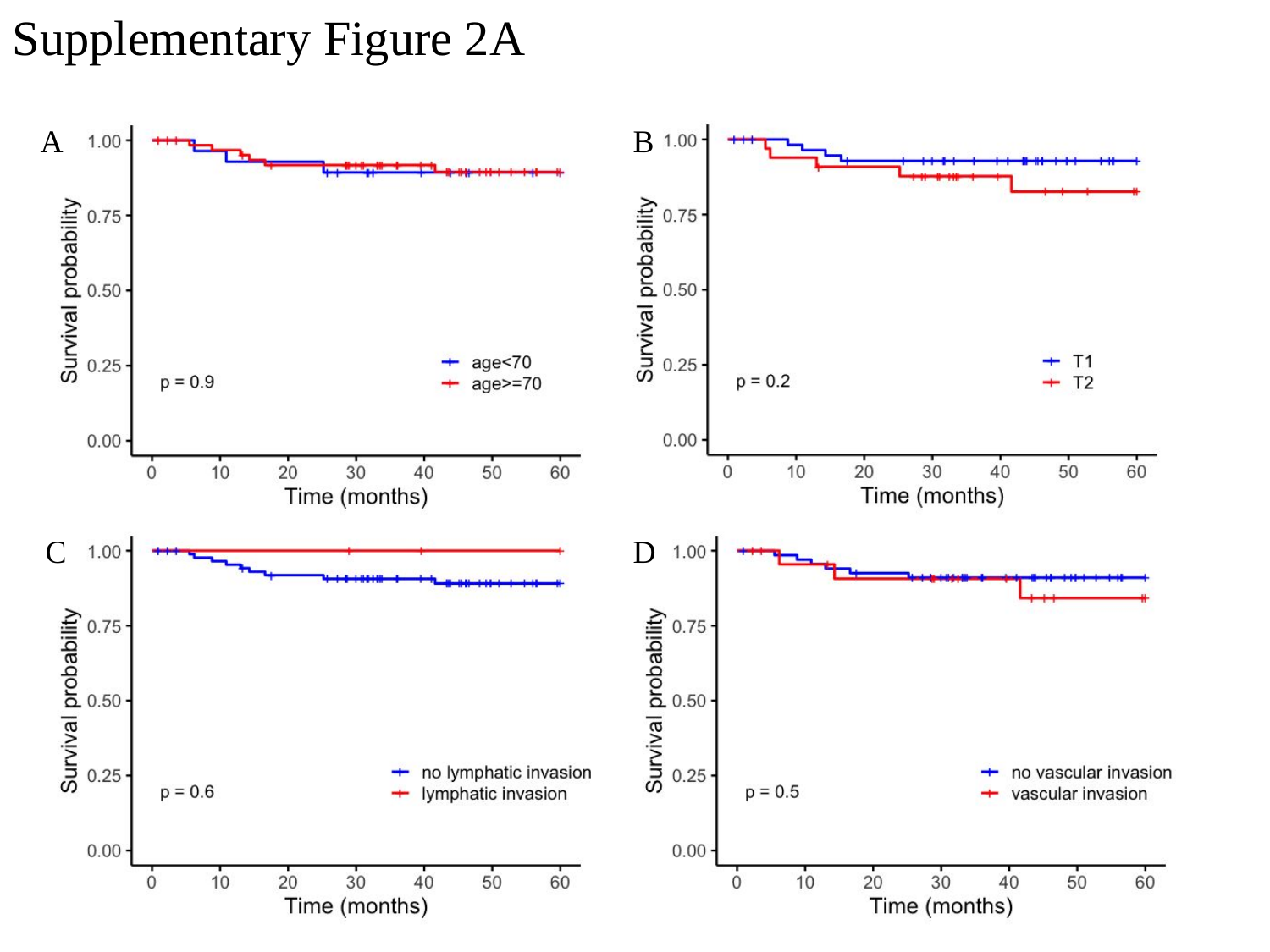

Supplementary Figure 2A
A
B
C
D

## Slide 5
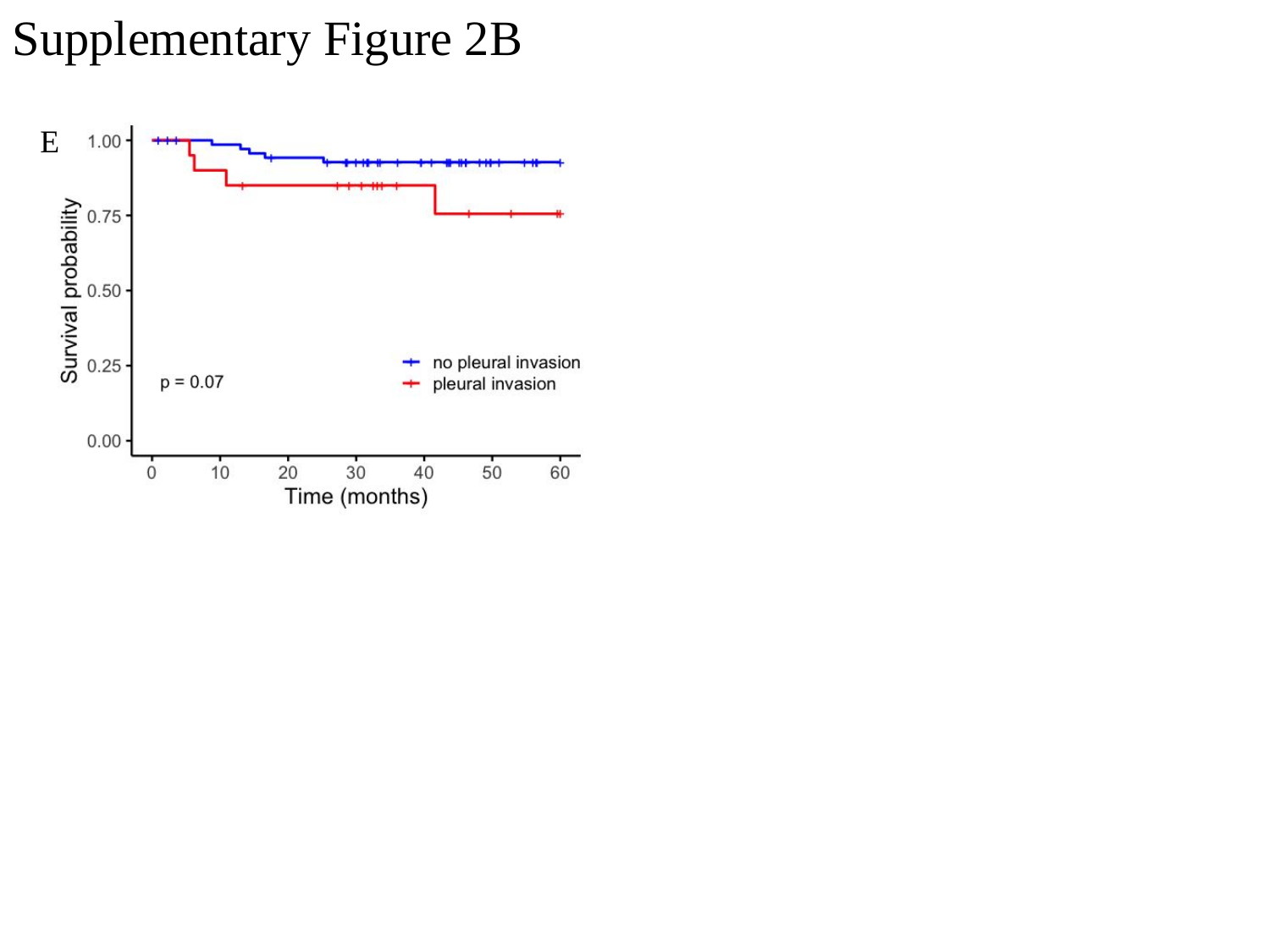

Supplementary Figure 2B
E

## Slide 6
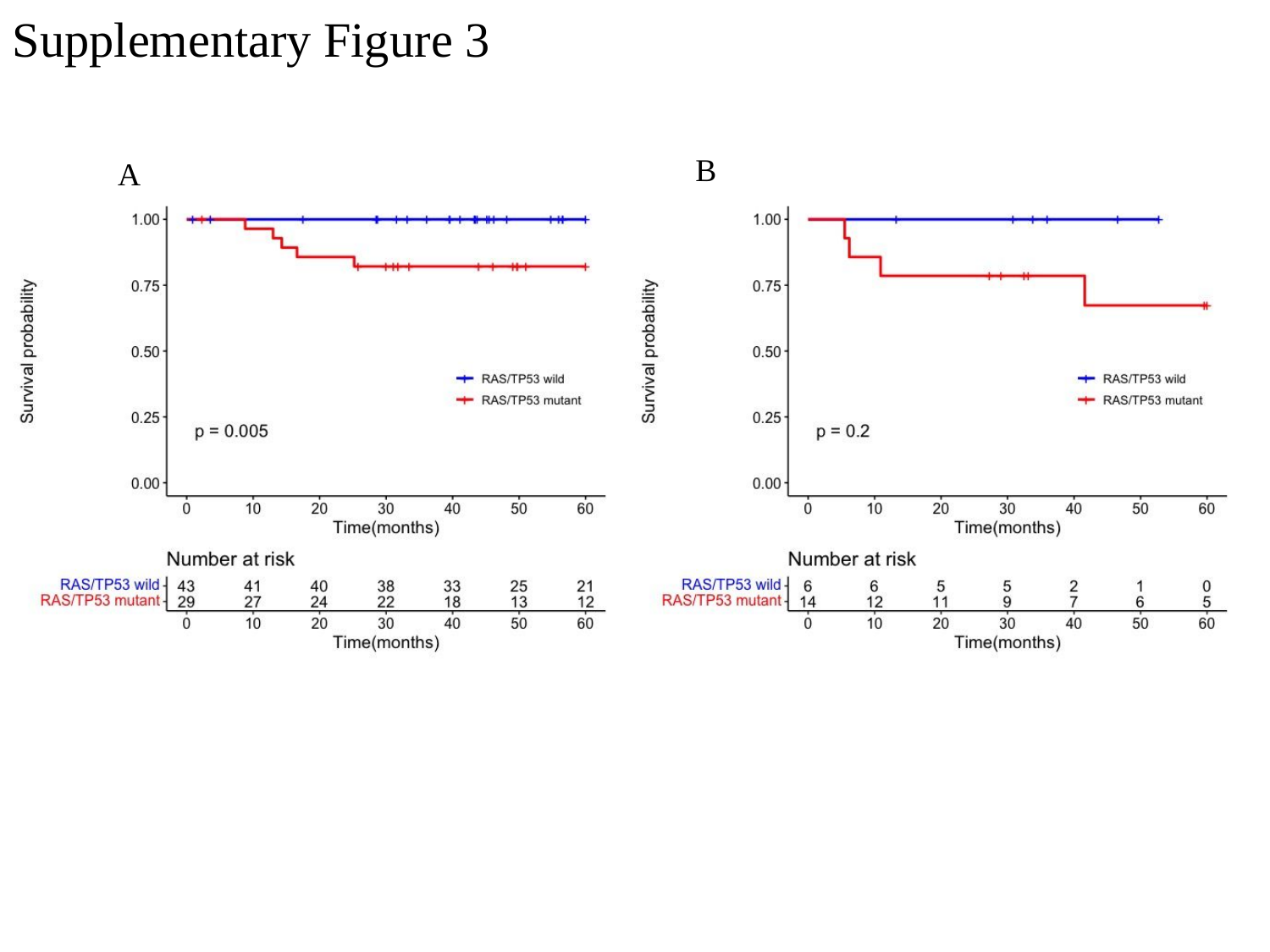

Supplementary Figure 3
B
A
