# Supplementary material for: The possibility of mutations of RAS signaling genes and/or TP53 in combination as a negative prognostic impact on pathological stage I non‐small cell lung cancer
Source: Cancer Med. 2023 Sep 15;12(19):19406–13. doi: 10.1002/cam4.6535 (PMC10587933; doi:10.1002/cam4.6535)
Supplement: Supplementary file 3 — Appendix S1 [file CAM4-12-19406-s002.docx]

**Supplementary Figure legends**

**Supplementary Figure 1A-C.** Relapse-free survival (RFS) in TMDU cohort stratified according to gene mutations; (1A) (A) *TP53*, (B) *KRAS*, (C) *EGFR*, (D) *PIK3CA*; (1B) (E) *CDKN2A*, (F) *NF1*, (G) *NFE2L2*, (H) *SMAD4*; (1C) (I) *STK11*.

**Supplementary Figure 2A-B.** Relapse-free survival (RFS) in TMDU cohort stratified according to candidate prognostic factors in ref 10; (2A) (A) Age, (B) T factor, (C) Lymphatic invasion, (D) Vascular invasion; (2B) (E) Visceral pleural invasion.

**Supplementary Figure 3.** Relapse-free survival (RFS) of visceral pleural invasion negative (A) and positive group (B) in TMDU cohort. RFS is stratified by RAS/TP53 wild type and mutant patients in each group.
